# Pacific Ocean–Wide Profile of CYP1A1 Expression, Stable Carbon and Nitrogen Isotope Ratios, and Organic Contaminant Burden in Sperm Whale Skin Biopsies

**DOI:** 10.1289/ehp.0901809

**Published:** 2010-12-06

**Authors:** Céline A.J. Godard-Codding, Rebecca Clark, Maria Cristina Fossi, Letizia Marsili, Silvia Maltese, Adam G. West, Luciano Valenzuela, Victoria Rowntree, Ildiko Polyak, John C. Cannon, Kim Pinkerton, Nadia Rubio-Cisneros, Sarah L. Mesnick, Stephen B. Cox, Iain Kerr, Roger Payne, John J. Stegeman

**Affiliations:** 1 Woods Hole Oceanographic Institution, Woods Hole, Massachusetts, USA; 2 Ocean Alliance, Lincoln, Massachusetts, USA; 3 Department of Environmental Toxicology, Institute of Environmental and Human Health, Texas Tech University, Lubbock, Texas, USA; 4 Department of Environmental Sciences, University of Siena, Siena, Italy; 5 Department of Biology, University of Utah, Salt Lake City, Utah, USA; 6 Botany Department, University of Cape Town, Cape Town, South Africa; 7 Instituto de Conservación de Ballenas, Buenos Aires, Argentina; 8 Centro de Investigationes Biologicas del Noroestre, La Paz, Mexico; 9 Southwest Fisheries Science Center, National Oceanic and Atmospheric Administration, La Jolla, California, USA

**Keywords:** biomarkers, CYP1A1, cytochrome P450, marine ecosystem, marine mammal, PAH, PCB, PHAH, sperm whale, stable isotope

## Abstract

**Background:**

Ocean pollution affects marine organisms and ecosystems as well as humans. The International Oceanographic Commission recommends ocean health monitoring programs to investigate the presence of marine contaminants and the health of threatened species and the use of multiple and early-warning biomarker approaches.

**Objective:**

We explored the hypothesis that biomarker and contaminant analyses in skin biopsies of the threatened sperm whale (*Physeter macrocephalus*) could reveal geographical trends in exposure on an oceanwide scale.

**Methods:**

We analyzed cytochrome P450 1A1 (CYP1A1) expression (by immunohistochemistry), stable nitrogen and carbon isotope ratios (as general indicators of trophic position and latitude, respectively), and contaminant burdens in skin biopsies to explore regional trends in the Pacific Ocean.

**Results:**

Biomarker analyses revealed significant regional differences within the Pacific Ocean. CYP1A1 expression was highest in whales from the Galapagos, a United Nations Educational, Scientific, and Cultural Organization World Heritage marine reserve, and was lowest in the sampling sites farthest away from continents. We examined the possible influence of the whales’ sex, diet, or range and other parameters on regional variation in CYP1A1 expression, but data were inconclusive. In general, CYP1A1 expression was not significantly correlated with contaminant burdens in blubber. However, small sample sizes precluded detailed chemical analyses, and power to detect significant associations was limited.

**Conclusions:**

Our large-scale monitoring study was successful at identifying regional differences in CYP1A1 expression, providing a baseline for this known biomarker of exposure to aryl hydrocarbon receptor agonists. However, we could not identify factors that explained this variation. Future oceanwide CYP1A1 expression profiles in cetacean skin biopsies are warranted and could reveal whether globally distributed chemicals occur at biochemically relevant concentrations on a global basis, which may provide a measure of ocean integrity.

Ocean pollution is an increasing economic and health concern worldwide, threatening resource populations and seafood safety [Intergovernmental Oceanographic Commission (IOC) 2001; Knap et al. 2002; National Research Council 2007; Pew Oceans Commission 2003; U.S. Commission on Ocean Policy 2004). Assessing oceanic health requires international multidisciplinary investigations at a scale and frequency difficult to manage logistically and financially. Measures of “ocean ecosystem integrity” identified by the Global Ocean Observing System (GOOS) and the IOC include the health of endangered and threatened species and the presence and impact of persistent organic pollutants such as planar halogenated aromatic hydrocarbons (PHAHs) and polycyclic aromatic hydrocarbons (PAHs) (IOC 2001; Knap et al. 2002). One of the recommended approaches to gather information on these measures, and therefore on ocean integrity, is the use of biomarkers such as the cytochrome P450 (CYP) enzymes (Knap et al. 2002).

Previous reports indicated that marine mammals may be sentinels for ocean and human health (De Guise et al. 1995; Dewailly et al. 1993; Fair et al. 2010; Fossi et al. 2003; IOC 2001; Moore 2008; O’Hara et al. 1999; Wells et al. 2004). Although pinnipeds can be effective coastal sentinels (Ross 2000), cetaceans may be better suited to risk assessment of oceanic systems because they do not rely on land or ice for survival. Large-scale assessments on long-lived vertebrate species may provide an important perspective on long-term impacts of environmental pollution by integrating multiple indirect influences, such as atmospheric and oceanic pollutant distribution or trophic interactions operating at wide geographical scales (Peterson et al. 2003; Tanabe et al. 1994).

Here, we examined biomarkers (CYP1A1 expression, stable isotope ratios) and chemical residues in the sperm whale (*Physeter macrocephalus*). We selected this species for its worldwide distribution, threatened status (Taylor et al. 2008), high position within food chains, and long life span, which allows for integration of long-term pollutant exposure. Additionally, its characteristic vocalization allows for acoustic detection and tracking. CYP1A1 is induced by agonists for the aryl hydrocarbon receptor (AHR) (Denison and Nagy 2003). Following GOOS and IOC recommendations, we examined the expression of CYP1A1 as a biomarker of exposure to analytes of concern (PHAHs and PAHs) and included sex determination, as well as stable isotope and contaminant burden analyses, in our investigation. Our study area encompassed five locations across the whole Pacific Ocean, including tropical/subtropical regions and small island states, areas suggested as priorities for human and ocean health assessment (Knap et al. 2002). Our objectives were to *a*) compare CYP1A1 expression among five Pacific Ocean regions; *b*) investigate whether sex and age, diet and trophic level, and movement and migration influenced regional CYP1A1 expression; and *c*) determine whether CYP1A1 expression correlated with pollutant exposure. To our knowledge, this is the first study to assess such broad geographical trends in CYP1A1 expression, stable carbon and nitrogen isotopes, and polychlorinated biphenyl (PCB) and other organic contaminant burdens at the ocean system level in a threatened cetacean species.

## Materials and Methods

### Biopsy collection

Sperm whale biopsies were obtained in the Gulf of California (GC), Mexico, in August–November 1999; Galapagos Islands (GP), Ecuador, in April–June 2000; Pacific waters between GP and Kiribati [Pacific Crossing 1 (PX1)] in July–August 2000; Kiribati (KR) in December 2000; and Papua New Guinea (PNG) in February–May 2001. We collected skin and blubber biopsies by crossbow and arrow under U.S. National Marine Fisheries Service permit 1004 to Ocean Alliance [for additional information on permits, see Supplemental Material (doi:10.1289/ehp.0901809)]. Biopsy arrows were fitted with collection tips 40 mm depth × 7 mm internal diameter. Only subadults or adults were sampled. Bulls (sexually mature males) were easily identified by size (Best 1979). Animals were treated humanely and according to federal biopsy sampling standards. Immediately after collection, each biopsy was divided into several subsamples, including a full longitudinal slice in 10% neutral buffered formalin for immunohistochemistry (IHC), and a skin subsample in 20% dimethyl sulfoxide solution saturated with NaCl for sex-typing analysis. Additional skin and blubber subsamples were stored at –20°C for stable isotope and contaminant analyses, respectively.

### IHC

We prepared samples for CYP1A1 IHC with the monoclonal antibody MAb 1-12-3, as previously described (Godard et al. 2004). MAb 1-12-3 is specific for CYP1A1 in mammals (Drahushuk et al. 1998) and has been shown to identify a single band in beluga whale (*Delphinapterus leucas*) liver microsomes after Western blotting (White et al. 1994). CYP1A1 expression was quantified as a staining score (0–15) calculated as the product of staining occurrence (0–3) and intensity (0–5) [Godard et al. 2004; see also Supplemental Material (doi:10.1289/ehp.0901809)]. This scoring technique accurately reflects CYP1A1 protein expression (Woodin et al. 1997). In addition, we estimated proxy CYP1A1 IHC scores for pooled samples from each region by summing individual sample IHC scores multiplied by the contribution of the individual section to the overall pooled sample weight. We evaluated tissue integrity (nuclear stain intensity, nucleus shape, eosinophilia) using tissue sections stained with hematoxylin and eosin to confirm that samples were adequate for IHC.

### Sex determination

We isolated skin DNA using a Fastprep tissue pulverizer (MP Biomedicals, Irvine, CA). Sex was determined by amplification of SRY (male determining factor) via multiplex polymerase chain reaction (Rubio-Cisneros et al. 2006).

### Analytical chemistry

#### Individual samples

We selected 10 samples from each region for individual sample assays, including four samples selected at random from samples within the highest (upper 33%) CYP1A1 IHC score group for each region, and three each from the median and lower third score groups for each region. These samples were analyzed for PCBs, dichlorodiphenyltrichloroethane (DDT), and hexachlorobenzene (HCB) according to U.S. Environmental Protection Agency Method 8081/8082, as previously described (Ballschmiter and Zell 1980) with modification (Marsili and Focardi 1996). We calculated the percentage of extracted organic material (%EOM) for each subsample after lyophilization and extraction with *n*-hexane. Small subsample sizes prevented further determination of lipid content in the EOM. Thirty PCB congeners were resolved [see Supplemental Material, Table 1 (doi:10.1289/ehp.0901809)] using a temperature gradient. Total PCB concentrations (∑PCBs) were quantified as the sum of all congeners analyzed. Sample size precluded separate analysis of dioxin-like PCBs. (Two mono-*ortho*-substituted PCBs, congeners 118 and 156, were detected but in conjunction with non-dioxin-like congeners.) Total DDT concentrations (∑DDTs) were calculated as the sum of *o*,*p*′-DDT, *p*,*p*′-DDT, *o*,*p*′-dichlorodiphenyldichloroethane (*o*,*p*′-DDD), *p*,*p*′-DDD, *o*,*p*′-dichlorodiphenyldichloroethylene (*o*,*p*′-DDE), and *p*,*p*′-DDE. Results are expressed in nanograms per gram EOM [for detailed methodology, see Supplemental Material (doi:10.1289/ehp.0901809)].

#### Pooled samples

We pooled all samples collected in each region by sex (eight pooled samples total because we collected only males in GP and females in KR) to obtain enough material to determine PAHs. We prepared pooled samples by combining complete vertical sections of blubber samples (section weight range, 3.7–184 mg); small sample sizes prevented pooling of sections of identical weight. PAHs were extracted according to Griest and Caton (1983) with modifications (Marsili et al. 2001) and analyzed by HPLC with fluorescence detection as described by Marsili et al. (2001). Results were expressed as nanogram of PAHs per gram of EOM for the sum of 15 PAHs [∑PAHs; naphthalene, acenaphthene, fluorene, phenanthrene, anthracene, fluoranthene, pyrene, benzo(*a*)anthracene, chrysene, benzo(*b*)fluoranthene, benzo(*k*)fluoranthene, benzo(*a*)pyrene, dibenzo(*ah*)anthracene, benzo(*ghi*)perylene, indeno(1,2,3-*cd*)pyrene; for details, see Supplemental Material (doi:10.1289/ehp.0901809)]. We also analyzed ∑PCBs, ∑DDTs, HCB, and %EOM in each set of pooled samples in relation to proxy CYP1A1 IHC scores for the pooled samples. Separate analysis of dioxin-like PCBs was precluded by small sample size.

#### Stable isotope analyses

One hundred skin subsamples were randomly selected among animals biopsied at locations where fish or squid were concurrently collected. Subsamples were dried, powdered, and analyzed using a Carlo-Erba 1108 elemental analyzer (Carlo-Erba, Milan, Italy) coupled to a Thermo Finnigan Delta-S isotope ratio mass spectrometer (ThermoFisher Scientific, Waltham, MA). Stable carbon and nitrogen isotope ratios are expressed as δ^13^C or δ^15^N (‰) [for details, see Supplemental Material (doi:10.1289/ehp.0901809)].

### Statistical analyses

CYP1A1 staining scores were not normally distributed, warranting nonparametric testing. We assessed geographic variation in CYP1A1, contaminant burden, and EOM using the Kruskal-Wallis rank sum test. We used pairwise Wilcoxon rank sum tests for post hoc evaluation and Holm’s sequential adjustment of *p-*values to control for error rates. We used Spearman’s rank correlation ρ to evaluate correlations among cell-specific CYP1A1 expression, EOM, and contaminant burden. We evaluated differences in CYP1A1 expression among sexes using the Wilcoxon rank sum test with continuity correction. We used one-way analysis of variance and Tukey-Kramer post hoc tests to evaluate variation in stable isotope signatures among sites and one-tailed *t*-tests to analyze whether ratios between two regions differed by set values. Analyses were conducted using R (R Development Core Team 2008); *p* < 0.05 was considered statistically significant.

## Results

**Figure 1:**
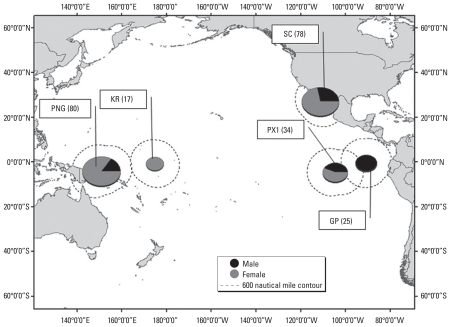
Locations of study sites, with whale numbers given in parentheses and sex ratios. Each chart’s center coordinates represent the mean of the highest and lowest latitudes and the mean of the most eastern and western longitudes of animals biopsied in that region. Chart size reflects sample size, and contours represent 600-nautical-mile buffer zones from location of whales sampled farthest from chart center in all directions.

We analyzed skin biopsies taken from sperm whales (*n* = 234) in five Pacific Ocean regions for CYP1A1 expression, sex, stable isotope ratios, and contaminant levels (Table 1). We sampled both sexes in GC, PX1, and PNG, but we encountered only males in GP and only females in KR (Figure 1). Overall, 68% of the animals biopsied were females. Sixteen males (three, six, four, and three from GC, GP, PX1, and PNG, respectively) were classified as bulls in the field, with subsequent laboratory confirmation by sex typing.

**Figure 2:**
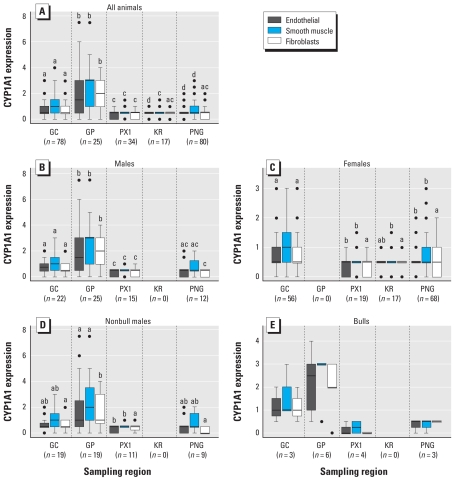
Cell-specific CYP1A1 expression in sperm whale skin biopsies among sampling locations. (*A*) All animals (*n* = 234). (*B*) All males (*n* = 74). (*C*) Females (*n* = 160). (*D*) Nonbull males (*n* = 58). (*E*) Bulls (*n* = 16). All GP animals were males, and all KR animals were females. Boxes represent 25th to 75th percentiles, lines within boxes indicate the median, whiskers represent minimum and maximum, and black circles indicate outliers. Different letters indicate statistically different means within a specific cell type (*p* < 0.05). Regional differences were detected in bulls (*p* < 0.05), but small sample size precluded statistical analysis and thus ranking of means.

We observed CYP1A1 staining in endothelial cells, smooth muscle cells, and fibroblasts, as reported previously (Godard et al. 2004). GP CYP1A1 scores were the highest of all sites and greater than the lowest regional scores (KR and PX1 scores) by factors of 4 and 5, respectively [Figure 2; see also Supplemental Material, Table 2 (doi:10.1289/ehp.0901809)]. CYP1A1 scores in GC were the second highest and significantly higher than those found in PX1, KR, and PNG for some cell types. We observed adequate tissue integrity for IHC assays in all samples examined and no staining in control slides incubated with nonspecific antibody.

CYP1A1 expression differed significantly among sites. When we considered all animals (Figure 2A) or only males (bulls and nonbull males combined; Figure 2B), GP animals had the highest levels of CYP1A1 staining in all cell types, and GC animals the second highest. We observed a similar pattern when we considered only nonbull males (Figure 2D) or only bulls (Figure 2E), but the small number of bulls precluded statistical analysis and thus ranking of IHC scores among regions. GP scores were significantly higher than PX1 scores in all cell types and higher than all other regional scores in fibroblasts in all analyses where sample size allowed ranking (Figure 2A,B,D). The GP and PX1 scores differed by a factor of at least 11 in bulls and by a factor of at least 4 in nonbull males. In females, CYP1A1 expression was highest in GC animals (no females were sampled in GP; Figure 2C).

We observed no differences in CYP1A1 expression levels between females and males (bulls plus nonbulls), or between females and nonbull males in regions where both sexes were sampled, whether they were analyzed by region or for all regions combined [see Supplemental Material, Figure 1 (doi:10.1289/ehp.0901809)]. The small number of bulls precluded comparisons between bulls and nonbulls in individual regions, but we observed no difference between bulls and nonbulls when all regions were combined.

**Figure 3:**
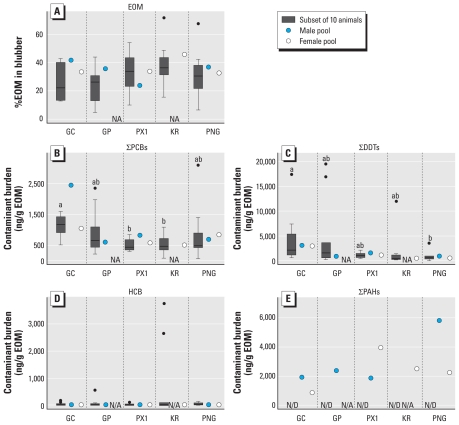
Contaminant burdens and %EOM in subsets of 10 animals and values in male/female pooled blubber samples from each region. NA, not applicable. (*A*) Percent EOM. (*B*) ∑PCBs. (*C*) ∑DDTs. (*D*) HCB. (*E*) ∑PAHs. Boxes represent 25th to 75th percentiles, lines within boxes indicate the median, whiskers represent minimum and maximum, and black circles indicate outliers. Different letters indicate statistically different means within a specific sample type (*p* < 0.05).

The %EOM and HCB burden in the 10-animal subsets, or in pooled male or female blubber samples, did not differ among regions (Figure 3A,D). ∑PCB, ∑DDT, and ∑PAH burdens were similar across regions in the pooled blubber samples (Figure 3B,C,E). The mean ∑PCB burden was significantly higher in the individual samples from GC than in corresponding samples from PX1 or KR (Figure 3B), whereas the mean ∑DDT burden was significantly higher in individual samples from GC than in PNG (Figure 3C). ∑PCB, ∑DDT, and HCB burdens in individual samples were positively correlated with %EOM, and ∑PCB burdens were positively correlated with ∑DDT and HCB burdens, but we found no correlation between ∑DDT and HCB burdens [see Supplemental Material, Table 3 (doi:10.1289/ehp.0901809)]. Individual CYP1A1 IHC scores (in all 50 animals or in males or females) were not significantly correlated with %EOM or any contaminant burden analyzed with the exception of HCB burden in endothelial cells in males (see Supplemental Material, Table 4). Proxy CYP1A1 IHC scores in pooled samples were not significantly correlated with %EOM, ∑PCB, or ∑PAH burdens in pooled samples, whether analyzed by sex or in both sexes combined (see Supplemental Material, Table 5).

**Figure 4:**
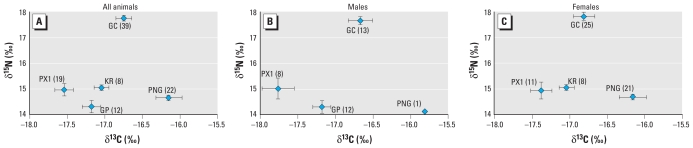
Stable isotope ratios in sperm whales from five Pacific Ocean sites. Ratios are expressed as mean ± 1 SE, with sample sizes given in parentheses. (*A*) All animals (*n* = 100). (*B*) Males (*n* = 34). (*C*) Females (*n* = 65). Sex typing was unsuccessful in one sample; that sample is included only in *A*.

None of the individual samples assayed for stable nitrogen and carbon isotope analyses were from bulls [Figure 4; see also Supplemental Material, Table 6 (doi:10.1289/ehp.0901809)]. Mean δ^15^N values were significantly higher in samples from GC than other regions, but values differed by < 3.5‰, 3.2‰, 2.9‰, and 2.8‰ for GP, PX1, KR, and PNG, respectively (Figure 4A). Mean GC δ^15^N values in males (Figure 4B) and females (Figure 4C) were also higher than in other regions, but differences were less pronounced than for all samples combined. Mean δ^15^N values did not differ significantly among samples from the equatorial Pacific (GP, PX1, KR, and PNG; range, 14.3–15.1‰). We analyzed stable isotope ratios because they provide general information on the whales’ exact trophic levels, which are unknown in our study. We could not calculate the whales’ trophic level directly because the required values for stable isotope ratios at the base of the regional food webs were unknown and because we did not collect sufficient fish/squid samples consistently in each region to estimate them.

Mean δ^13^C did not differ significantly among whales sampled in GP, PX1, and KR [range, −17.5‰ to −17.1‰; Figure 4A; see also Supplemental Material, Table 6 (doi:10.1289/ehp.0901809)]. GC whales had significantly higher mean δ^13^C values than did PX1 whales, and PNG whales had significantly higher mean δ^13^C than GP, PX1, and KR whales, but values differed by < 1.1‰ (PNG and PX1) or by < 1‰ (all others). In males (Figure 4B), δ^13^C was higher in GC than in PX1, and in females (Figure 4C), δ^13^C values were higher in PNG than in other regions, but mean values differed by < 1‰.

## Discussion

### Dermal CYP1A1 staining

In the present study we measured dermal CYP1A1 expression in sperm whales from a geographic range encompassing the breadth of the Pacific basin, providing a unique baseline for global assessment of exposure biomarkers. Dermal CYP1A1 expression was detected by IHC, a robust method advocated for cetacean biopsies (Angell et al. 2004; Wilson et al. 2007). We previously reported that CYP1A1 was induced in sperm whale skin biopsy slices in a dose-dependent manner by the AHR agonist β-naphthoflavone (Godard et al. 2004). The usefulness of dermal CYP1A1 expression as a biomarker of PHAH and PAH exposure in cetaceans is further supported by studies showing that CYP1A1 levels or activity in skin and liver correlate with blubber PCB concentrations in cetaceans (Fossi et al. 2003; Marsili et al. 1998; White et al. 1994; Wilson et al. 2007), and oral exposure to inducers elicits a dose-dependent induction of dermal CYP1A1 activity and protein in aquatic and terrestrial mammals (Ben-David et al. 2001; DeVito et al. 1997).

We detected significant regional differences in CYP1A1 expression and subsequently examined the potential confounding effects of age, sex, and trophic level, the primary factors influencing contaminant accumulation in cetaceans (Boon et al. 1992; Borrell et al. 1995). An organism’s trophic level refers to the position it occupies in the food chain. Additional factors known to influence CYP1A1 expression were also considered. One study in wild bottlenose dolphins (*Tursiops truncatus*) reported that dermal CYP1A1 expression was not influenced by age, sex, or reproductive status but was correlated with PCB exposure (Wilson et al. 2007). To our knowledge, little else has been reported on these potential confounding factors in wild cetaceans, where age, sex, and health are rarely known.

### Potential confounding effects of sex and age

Reproductively active female marine mammals tend to have lower chemical burden loads than their male counterparts because of lactational transfer of lipophilic contaminants (Borrell et al. 1995). Reproductively immature whales of both sexes from the same area are likely to have similar exposures. However, no correlation between pollutant burden and age was reported in juvenile or mature sperm whales of both sexes that mass stranded in Tasmania (Evans et al. 2004), and neither age nor sex influenced CYP1A1 expression in skin biopsies of Florida wild bottlenose dolphins (Wilson et al. 2007). In the present study, the highest and second highest regional CYP1A1 expression levels were detected in GP and GC whales, respectively. This was true whether we included all animals, only males, or only females in the analyses. In addition, CYP1A1 scores between females and nonbull males and between females and bulls did not differ in regions where both sexes were sampled, further suggesting that sex may not have been a confounding factor in our study. We compared CYP1A1 levels between bulls and nonbull males to further ascertain whether regional differences might be driven by older males, which could accumulate greater amounts of contaminants over the long times in polar regions where they reside when not breeding. We found no significant differences in CYP1A1 scores between bulls and nonbull males when all regions were combined. The sample size for bulls was too small to allow comparison in individual regions. However, we observed similar regional trends whether bulls and nonbull males were examined individually or together. Based on these results, we cannot conclude that sex or the presence of bulls drove the regional variation observed in CYP1A1 staining.

### Potential confounding effects of diet and trophic level

Sperm whales are considered dietary specialists that feed on a narrow range of prey, but diet composition may differ with location and season, and bulls may feed on larger prey than do females (Whitehead 2003). Using stable isotope analyses, we examined whether potential variation in diet could have resulted in trophic level differences. Marine vertebrates are significantly enriched in ^15^N relative to their prey, and a difference of 3.6‰ or more between two species can indicate one trophic level increase (Fry 1988), that is, a higher position within the food chain that may lead to increased pollutant biomagnification in one species. Sperm whale δ^15^N values did not differ across the equatorial Pacific (GP, PX1, KR, and PNG), whereas GC δ^15^N values were higher than in other regions but by < 3.6‰, suggesting similar trophic positions at time of sampling. Furthermore, the difference between GC and GP δ^15^N values was the closest to the 3.6‰ cutoff, and yet GP CYP1A1 scores were significantly higher than those of GC. If GC whales had fed on higher trophic level prey than had GP whales, we might have predicted higher CYP1A1 scores in GC than in GP whales. Hence, the stable isotope data suggest that trophic level did not contribute to regional differences in our study, although potential seasonal/annual variation in stable isotopes cannot be ruled out, and the whales’ specific trophic level could not be calculated. Overall, detailed foraging data identifying prey species, size, and trophic levels are poorly known or unavailable for sperm whales in many areas within their worldwide range, including PX1, KR, and PNG. Nevertheless, based on our analyses, it appears that trophic level did not drive the regional variation observed in CYP1A1 scores.

### Potential confounding effects of movement and migration

Although sperm whale population movements within ocean basins are largely unknown, a 1,000-km (< 600 nautical mile) range over 10 years has been estimated for females and juveniles (Dufault et al. 1999). Reports of females traveling longer distances and between GP and GC exist (Jaquet et al. 2003). However, a detailed study of long- distance movements in 3,889 sperm whales in the tropical Pacific from 1985 to 2004 confirmed an average 1,000 km displacement in females and juveniles after a period of ≥ 1 year and showed movement between GP and GC to be rare (Whitehead et al. 2008). Based on a 1,000-km range, only GP and PX1 whales in our study had potential geographic overlap. GP CYP1A1 staining scores were the highest and PX1 among the lowest, suggesting that whale movement is unlikely to have driven the observed CYP1A1 regional trends. In addition, regional carbon isotope ratios were similar or differed by < 1.1‰. A difference > 1‰ between species can indicate a difference in sources of primary production and hence a difference in latitude (Ruiz-Cooley et al. 2004). The weak δ^13^C variation we observed suggests that the samples were collected from whales residing in similar latitudes. Overall, we conclude that the observed regional CYP1A1 scores are likely to reflect exposure of whales primarily within the study areas, although movement between sites cannot be ruled out unequivocally.

### CYP1A1 expression and contaminant analyses

We analyzed eight sex-specific pooled samples for HCB and ∑PCB, ∑DDT, and ∑PAH burdens and found no significant geographical variation. Other than our data, information on organochlorine and PAH contamination in marine mammals and other species in the remote sites we explored is scarce. Long-range atmospheric and oceanic transport is likely the major source of exposure (Tanabe et al. 1994). The HCB and ∑PCB, ∑DDT, and ∑PAH burdens measured in our samples are comparable to those reported in sperm whales and other cetacean species worldwide (Marsili et al. 2001; see also review tables presented by Aguilar et al. 2002; Aono et al. 1997; Evans et al. 2004), although differences in analytical techniques and burdens reported in either lipid or wet weight make comparisons problematic.

We found no significant correlations between proxy CYP1A1 IHC scores and ∑PCBs or ∑PAHs in pooled samples. Besides the small number of pooled samples (*n* = 8 pools), there are several possible explanations for this lack of correlation. First, proxy CYP1A1 scores may not reflect accurately CYP1A1 expression in the pooled samples. Second, because of the small size of blubber samples, we could detect only two dioxin-like PCBs (congeners 118 and 156), and we did not analyze other CYP1A1-inducing PHAHs. Thus, our ∑PCB data may not reflect the dioxin-like PCB burden present in our samples. Third, variation in contaminant concentrations with blubber depth has been observed in some cetacean species, including the sperm whale (Evans et al. 2004). Because of the small size of our biopsy tip (40 mm depth) relative to the average blubber thickness in sperm whale [98.4 ± 18.4 mm (mean ± SD); Evans et al. 2003], we consistently biopsied the outer blubber layer, which is less metabolically active than deep blubber. Stratification of CYP1A1 expression has been reported in blubber samples from some populations of bottlenose dolphins (Montie et al. 2008; Wilson et al. 2007). Interestingly, in one population, a weak correlation between ∑PCBs and endothelial CYP1A1 expression was observed in the deeper blubber but not the outer blubber (Wilson et al. 2007). Whether stratification of contaminant burden and CYP1A1 expression occurs generally in cetacean species is unknown and remains to be carefully investigated.

We analyzed individual samples from subsets of animals sampled from each region for ∑PCBs, ∑DDTs, and HCB. Animals included in each subset were selected from the upper, median, and lower third of the CYP1A1 IHC score groups in order to maximize chances of observing a potential correlation between CYP1A1 expression and contaminant concentration over a wide range of scores. However, we observed a significant correlation only between mean endothelial CYP1A1 score and HCB burden in males. HCB is a known partial AHR agonist able to weakly induce CYP1A (Hahn et al. 1989; Mundy et al. 2010). We observed large SEs in the analyses, especially for ∑DDT and HCB burdens, which could reflect the not totally random sample selection in the 10-animal subsets. This suggests that larger sample sizes would have been required to estimate means with more reasonable precision.

Overall, we did not observe a correlation between CYP1A1 scores and ∑PCBs in individual samples, or between proxy CYP1A1 scores and ∑PCBs or ∑PAHs in our sex-specific pooled blubber samples. However, limitations due in part to the small size of the blubber samples, including our inability to analyze most dioxin-like PCBs, the need to use proxy CYP1A1 scores for pooled samples, and the fact that we did not analyze other CYP1A1 inducing contaminants such as dioxins and furans prevent us from making any strong inferences regarding CYP1A1 expression as a correlate of regional contaminant burden.

Differential exposure to natural AHR agonists could contribute to the regional differences in expression of CYP1A1 in sperm whales. Ultraviolet light exposure can form tryptophan oxidation products that are potent AHR agonists (Wincent et al. 2009). Natural halogenated aromatics detected in marine mammal blubber (Teuten et al. 2005) are weak AHR agonists (Hahn M, personal communication), but their relative potency in vascular tissue cells has not been determined. The potential exposure and influence of these or other natural AHR ligands (e.g., volcanic PHAHS/PAHs, some algal toxins) on CYP1A1 expression in sperm whales warrants further research, perhaps especially in GP, where both anthropogenic and natural sources of AHR inducers are poorly known.

## Conclusions

We observed significant differences in levels of CYP1A1 expression in biopsy samples of sperm whales from different regions across the breadth of the Pacific Ocean. The highest levels were detected in whales from GP, a United Nations Educational, Scientific, and Cultural Organization World Heritage marine reserve, and the lowest CYP1A1 scores were detected in whales from the sampling sites farthest away from continents. Differences in age, sex, and diet did not appear to explain regional differences in CYP1A1 expression in our samples. With the exception of one association between HCB and CYP1A1 score in endothelium of males, we observed no significant correlations between the contaminant burdens analyzed and CYP1A1 expression. However, these analyses were limited by the small size of the blubber samples and did not include dioxin-like PCBs, dioxins, or natural products that are possible CYP1A1 inducers. Nonetheless, the regional differences in CYP1A1 expression and the contaminant analyses in our Pacific basin–wide study provide a unique baseline for global assessment of this biomarker, and possibly other molecular markers of exposure, susceptibility, or effect in sperm whale, a globally distributed and threatened species.

## Correction

In Figure 2 of the manuscript originally published online, data for GC were shown as GP data and vice versa, and data for PX1 were shown as KR data and vice versa. These errors have been corrected here.

## Figures and Tables

**Table 1:** Sample information.

Collection region (abbreviation)	Region boundaries (latitude and longitude)	Biopsies analyzed for CYP1A and sex (*n*)	Sex [M/F (% female)]	Biopsies analyzed for stable isotopes ratios (*n*)	Biopsies analyzed individually for ∑PCBs, ∑DDTs, and HCB levels (*n*)
Gulf of California (GC)	25°49.1′N to 28°24.4′N 110°03.3′W to 112°32.2′W	78	22/56 (72%)	39	10
Galapagos (GP)	01°20.8′S to 00°32.6′N 89°27.0′W to 92°03.9′W	25	25/0 (0%)	12	10
Pacific Crossing 1 (PX1)	03°55.8′S to 05°04.2′S 102°06.2′W to 107°13.0′W	34	15/19 56%	19	10
Kiribati (KR)	00°00.1′S to 01°17.1′S 173°43.3′E to 175°38.6′E	17	0/17 (100%)	8	10
Papua New Guinea (PNG)	02°17.2′S to 05°52.5′S 147°06.1′E to 154°24.9′E	80	12/68 (85%)	22	10
All sites	NA	234	74/160 (68%)	100	50

NA, not applicable.
